# Characterization of Sodium and Potassium Nitrate Contaminated Polyaniline-Poly (Ethylene Oxide) Composites Synthesized via Facile Solution Casting Technique

**DOI:** 10.3390/ma12132168

**Published:** 2019-07-05

**Authors:** Salma Bilal, Muhammad Arif, Muhammad Saleem Khan, Anwar-ul-Haq Ali Shah

**Affiliations:** 1 National Centre of Excellence in Physical Chemistry, University of Peshawar, Peshawar 25120, Pakistan; 2 TU Braunschweig Institute of Energy and Process Systems Engineering, Franz-Liszt-Straße 35, 38106 Braunschweig, Germany; 3 Institute of Chemical Sciences, University of Peshawar, Peshawar 25120, Pakistan

**Keywords:** polyaniline, poly (ethylene oxide), NaNO_3_, KNO_3_, composite, rheology

## Abstract

Fabrication of composites by developing simple techniques can be an effective way to modify some properties of individual materials. The present study relates to facile synthesis of sodium nitrate (NaNO_3_) and potassium nitrate (KNO_3_) contaminated polyaniline (PANI) and poly (ethylene oxide) (PEO) composites without using any additives, plasticizers, or fibers. The physic-chemical and rheological properties of synthesized composites were analyzed. The composites showed enhancement in both storage and loss modules in comparison with the polymer matrices. The dynamic viscosity of the synthesized materials has inverse relation with that of temperature and shear stress. Rheological analysis reveals a continuous drop off in viscosity by increasing shear stress. The flow behavior was affected little by temperature. However, the overall results showed a shear thinning effect suggesting that polymer composites show non-Newtonian behavior. The addition of NaNO_3_ and KNO_3_ had a profound effect on shear viscosity of the materials, although the overall shear thinning behavior prevails. The PANI-PEO composite follows, as the first approximation models, both Bingham and modified Bingham models, while the salt contaminated system follows only the Bingham model. Both show shear stress values. The greater values of storage (G′) and loss (G″) modulus of composites than PANI-PEO blend suggests excellent elasticity, better stiffness, and good mechanical strength of the composites. Furthermore, the composites were more thermally stable than pure polymers.

## 1. Introduction

Polyaniline (PANI) is considered as the first choice of researchers for various technological applications, such as in rechargeable batteries, sensors, anticorrosive coatings, switchable membranes, and electronic devices, due to its ease of synthesis, controlled electrical conductivity, excellent environmental stability [1,2]. However, it shows certain marked limitations which restrict its large scale applications. The main problems associated with PANI are considered to be its insolubility in common organic solvents, inability to process by conventional methods, and weak mechanical properties. The problem of insolubility has been effectively addressed in recent years by developing different strategies ultimately leading to the synthesis of soluble PANI [3,4,5,6]. Mixing of PANI with different conventional polymers/materials to form blends or composites can be the easiest and most facile method of improving its mechanical properties [7,8]. The blend or composite is generally a mixture of polymer with one or more materials which have relatively dissimilar physical or chemical properties [9]. This mixing can help in improving the electronic, mechanical, as well as the thermal properties in comparison with pure polymer [8]. PANI composites with various materials are used for a number of applications such as batteries, super capacitors, chemical sensors, electrochromic displays electric devices, in capacitors, and many more [2]. However, its blending or composite formation with conventional synthetic polymers still remains a challenge.

Expended graphite (EG) [10] and various polymers like polyvinyl alcohol (PVA) [11], poly (acrylonitrile-butadiene-styrene) [12], nafion [13], chitin [14], polystyrene and poly (ethylene oxide) [15] etc. can be useful to form composites with PANI [16] if PANI itself is soluble.

In recent years we have developed sophisticated methodologies for the synthesis of highly soluble and conducting PANI [4,5]. Once a soluble PANI is obtained, it becomes easy to modify its different properties via composites formation with other materials. 

In this context, the present study aims to fabricate composites of PANI with poly (ethylene oxide) (PEO) which is the well reputed member of the class of conventional polymers. The selection of PEO in this study is based on its remarkable properties, such as a low glass transition temperature, excellent flexibility, and the most important its ease complexation with different polymers (like PANI) and various salts which are unattainable by another polymer host.

PEO, being a polyether, has a strong tendency to make a hydrogen bond with PANI. The bond is established between ether oxygen of PEO, amine, or imine hydrogen of PANI while making its composites. PEO also makes a hydrogen bond with water molecules present in the PANI backbone. In a similar way, different active sites in PANI-PEO composites are responsible for accommodating the cation and anion of the potassium and sodium salts, which leads to the formation of new chemical bonds in the composite system. The bonding tendency of PEO depends upon the molecular weight, structural composition, orientation, and concentration in composites. PANI in this regard is considered an excellent candidate for PEO due to availability of different active sites to make a composite with PEO. The complexation in the present study is observed from the Fourier Transform Infra RED (FTIR) and X-rey Diffraction (XRD) results of the composite system.

**Scheme 1:**
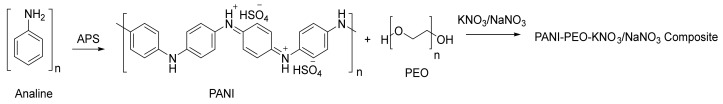
Preparation of PANI-PEO-KNO_3_/NaNO_3_ composites.

The schematic representation to elaborate the active sites and free functionalities of the polymers for counter parts of salts is presneted below in Scheme 1. 

The synthesized materials were characterized with different techniques and special emphasis was given to rheological studies, as such studies on PANI or PANI composites is very rare and can be helpful for some application purposes. The shear rate, shear stress behavior, effect of temperature, and change in viscoelastic properties of the prepared composite materials were determined. The synthesized composite materials were found thermally stable, elastic, and having high capability of storage. Therefore, these composites could be used as a good alternative as thermal protective coatings, in energy storage devices, and could be best suited for application in mechanically tough membranes.

## 2. Material and Methods

### 2.1. Materials

Research grade aniline (Acros, Schwerte, Germany) was distilled under vacuum and stored in nitrogen atmosphere. Chloroform (Sharlu, Wollongong NSW, Australia), dodecylbenzenesulfonic acid, (DBSA) and benzoyl peroxide (Merck, Kenilworth, NJ, USA), 2-butanol (Aldrich, St. Louis, MO, USA), poly (ethylene oxide) (PEO) and pyridine (molecular mass 400,000, BDH), NaNO_3_ and KNO_3_ salts (Sharlu) were used as received.

### 2.2. Methodology

The composites of PANI with PEO and nitrate salts of potassium and sodium were prepared in two phases. The first phase comprised the synthesis of PANI by inverse emulsion polymerization method as reported earlier [4]. The prepared PANI was mixed with PEO, NaNO_3_, and KNO_3_ with a solution casting technique using a chloroform and pyridine mixture as a solvent.

#### 2.2.1. Synthesis of PANI

In a typical experiment 35 mL chloroform was added to a 100 mL round bottom flask. 0.303 g of benzoyl peroxide was poured into the flask with constant mechanical stirring. 10 mL 2-butanol, 1.2 mL DBSA and 0.07 mL aniline were then added stepwise followed by addition of 5 mL of distilled water. A milky color solution was observed by addition of water. This mixture was stirred for 24 h. The end product of the reaction was separated into two layers in a separating funnel. The greenish organic phase solution having PANI was separated and cast in a petri dish for 24 h to dry. The dry product was washed by acetone several times until the undesirable products were removed by filtration, and then put in a desiccator for dryness. The dry flack film was formed and scratched by spatula in the form of powder.

#### 2.2.2. Synthesis of PANI-PEO-KNO_3_ and PANI-PEO-NaNO_3_ Composites

0.027 MPANI in chloroform was taken in a round bottom flask to form solution “A”. 0.025 M PEO (Mw 400,000) in chloroform was taken in another flask and termed as solution “B”. Similarly 0.1 g of potassium nitrate (KNO_3_) or sodium nitrate (NaNO_3_) was mixed with pyridine for solution “C”. It is worth noting that selection of solvent is an important factor while making composites. The sodium and potassium salts were completely miscible in pyridine while making solution “C”.

Each solution was stirred separately for a period of 4 h, and then they were mixed together. The mixture was stirred for 10–12 h at ambient temperature to get the homogeneous mixture. Then the mixture was cast on a petri dish to evaporate solvent (chloroform, pyridine) at room temperature. A jelly-like texture was throughout observed for the materials at room temperature. The final weight percent composition of the composites comprises of 50 wt% PANI, 40 wt% PEO, and 10 wt% salts.

#### 2.2.3. Characterization

Fourier-transform infrared spectroscopy (FTIR) analysis of the materials was carried out in the range of 400–4000 cm^−1^ by using aIRAffinity-1S Shimadzu Fourier Transform Infrared Spectrophotometer (Shimadzu, Tokyo, Japan). XRD (X-ray diffractometer) patterns of PANI were achieved by using Cu Kα radiations (λ = 1.5405 Å) JEOL JDX-3532 (JEOL, Tokyo, Japan). Scanning electron microscopy (SEM) micrographs were taken by using a JSM-6490 (JEOL, Tokyo, Japan) electron microscope. Thermal stability of PANI samples were determined by using Perkin Elmer, Diamond series (Waltham, MA, USA) at heat rate of 10 °C/min under nitrogen atmosphere. Electrochemical properties were studied by ALS/DY 2323 Biopotentiostate (ALS, Tokyo, Japan). For all these measurements, the samples were dried in vacuum. Rheology studies were carried out with the jelly-like texture of the materials by using Anton Paar Rheometer Physica MCR 301 (Graz, Austria) having a Peltier temperature control system which makes sure that no solvent evaporation takes place during the measurements.

## 3. Results and Discussion

### 3.1. FT-IR Spectroscopy

**Figure 1:**
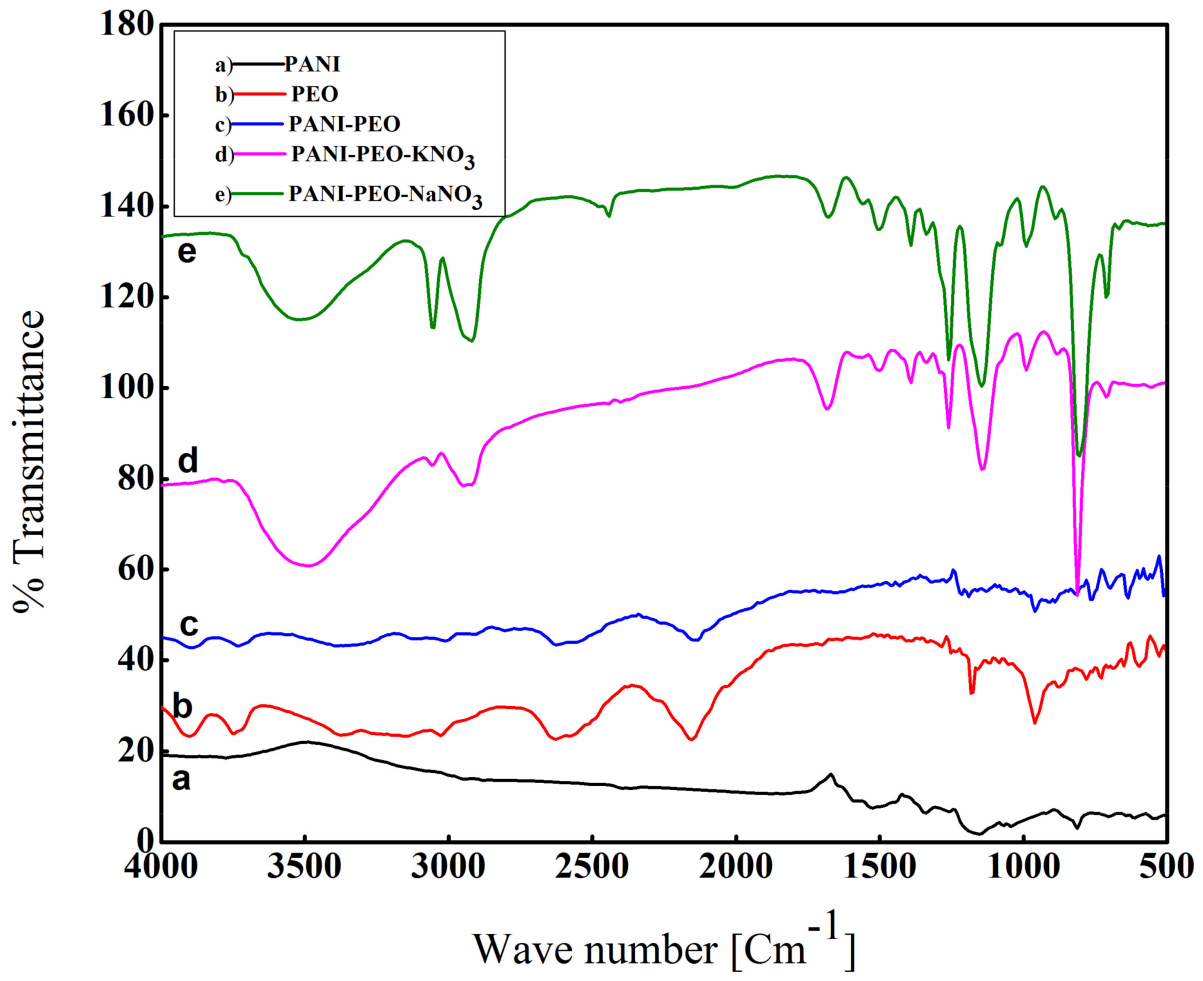
Spectra of (**a**) PANI (**b**) PEO (**c**) PANI-PEO (**d**) PANI-PEO-KNO_3_ and (**e**) PANI-PEO-NaNO_3_.

FT-IR spectroscopy was carried out in order to confirm the composite formation. The spectra are presented in Figure 1. The presence of 1548 cm^−1^and 1483 cm^−1^ bands in Figure 1a are attributed to the C=C stretching vibration inquinoid and benziod of the aromatic rings in PANI, respectively [17]. Figure 1b,c specify, respectively, FT-IR spectrum of pure PEO and PANI/PEO composite. A small peak at 1268 cm^−1^ shows the linking of C–N in the stretching mode. This implies the formation of new chemical bonds between PANI and PEO. The intensity of the peaks in the range of 2100 and 4000 cm^−1^, with reference PEO, is low [18]. Numerous aspects such as ring stretching, contraction of bonds in the ring and interactions of stretching modes might be involved in the compression of the peak areas of the composites [19].

FT-IR bands of the composites of PANI-PEO with KNO_3_ and NaNO_3_ salts (Figure 1d,e, respectively) show the primary constituent of the elements taking part in the composites formation. The band at 3490 cm^−1^ and 3510 cm^−1^ in Figure 1d,e verify the presence of N–H stretching of amine group of the PANI. Furthermore, the symmetric and asymmetric stretching vibration of C–H of the aromatic ring in the composites is assigned to the bands at 3010 cm^−1^, 3006 cm^−1^, 2900 cm^−^1, and 2885 cm^−1^ [20]. The three very distinguishing peaks in Figure 1d at 1221 cm^−1^, 1105 cm^−1^ and in Figure 1e at 1210 cm^−1^ and 1102 cm^−1^, describe the characteristics features of SO_4_ of DBSA, amine plus imine nitrogen. The bands 762 cm^−1^ in both composites indicate para substitution of aromatic rings of PANI [20] suggesting that the free functionalities of PANI have been occupied by KNO_3_ and NaNO_3_.

### 3.2. Thermal Properties

**Figure 2:**
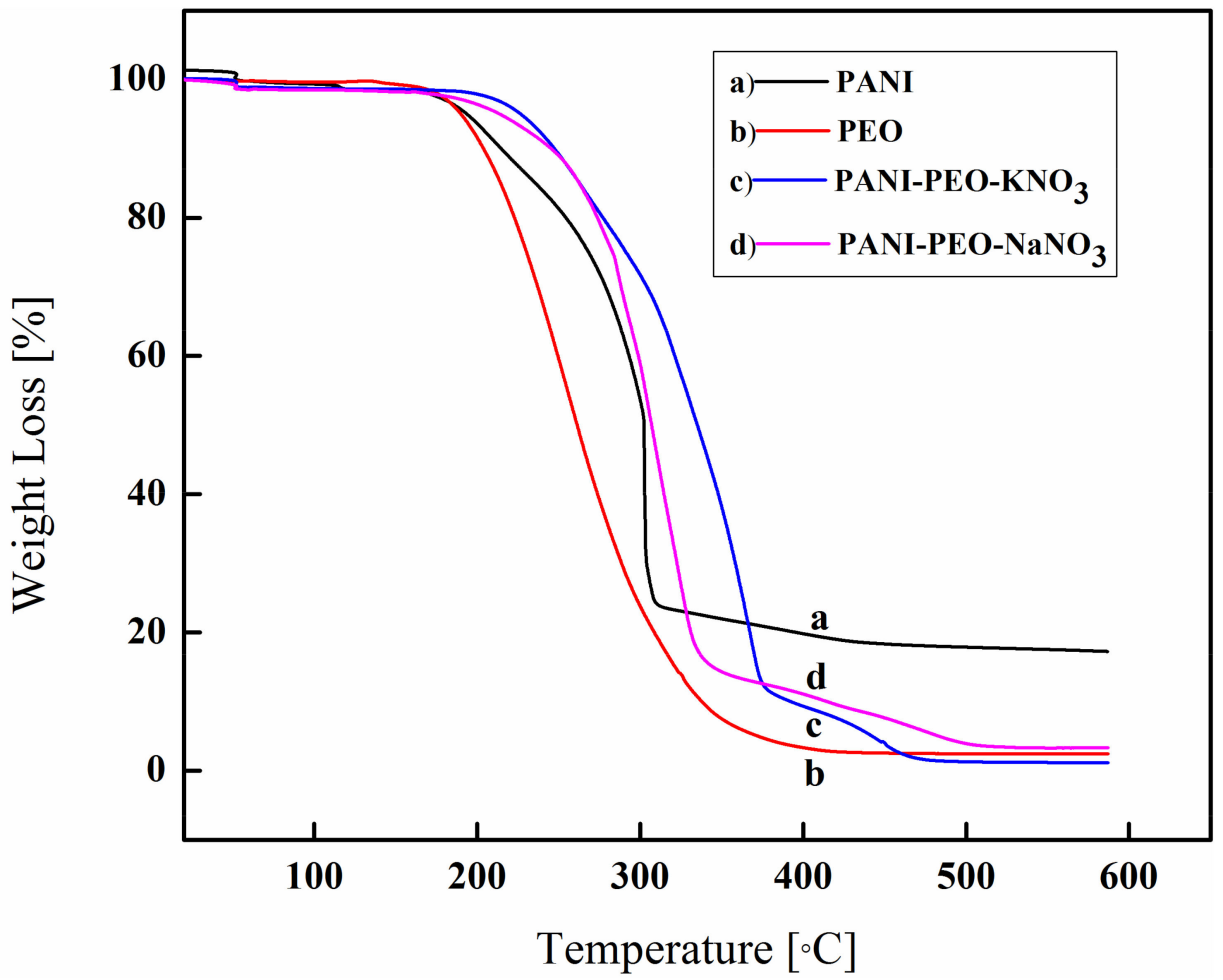
Curves for (**a**) PANI (**b**) PEO (**c**) PANI–PEO–KNO_3_ (**d**) PANI–PEO–NaNO_3_.

Figure 2a–d represent the thermal profile of PANI, PEO and its composites with KNO_3_ and NaNO_3_, respectively. It can be observed that the thermal stability of the polyaniline is enhanced by making a blend with PEO and nitrate salts of potassium and sodium. At 195 °C the first weight loss starts in PANI whereas 80–85% loss of the total weight happens up to 320 °C which shows the removal of DBSA from quinoid PANI ring. Similarly, the rapid weight loss comes into view after 320 °C which may be due to the sulphonate cluster in the DBSA. The remaining weight loss is due to the complete degradation of quinonoid ring structure [16]. 

Figure 2b shows that PEO is thermally degraded in a single uniform step. The mass remains stable up to 190 °C. The weight loss starts at 192 °C and shows complete degradation at about 360 °C, which is approximately 92% of the total mass. The remaining 8% is the carbonized form of PEO. Figure 2c,d signify two steps thermal stability of PANI-PEO-KNO_3_ and PANI-PEO-NaNO_3_ composites with thermal stability of 350–370 °C, respectively. The onsets as well as the offset decomposition temperature of the composites are higher than PANI counterparts. It is observed from the curves c and d that thermal properties of the composites have been enhanced. The inter-chain interaction between PANI and PEO affects the electron density and bond energy of PANI and hence the net result is the increase in the stability of the composites. Furthermore, the electrovalent type interaction between biphasic PANI-PEO and the inorganic salts may also be a possible cause of the enhanced thermal stability of tri-phase PANI-PEO-salts composites. The thermal stability of the synthesized materials is summarized in Table 1.

### 3.3. Scanning Electron Microscopy

**Figure 3:**
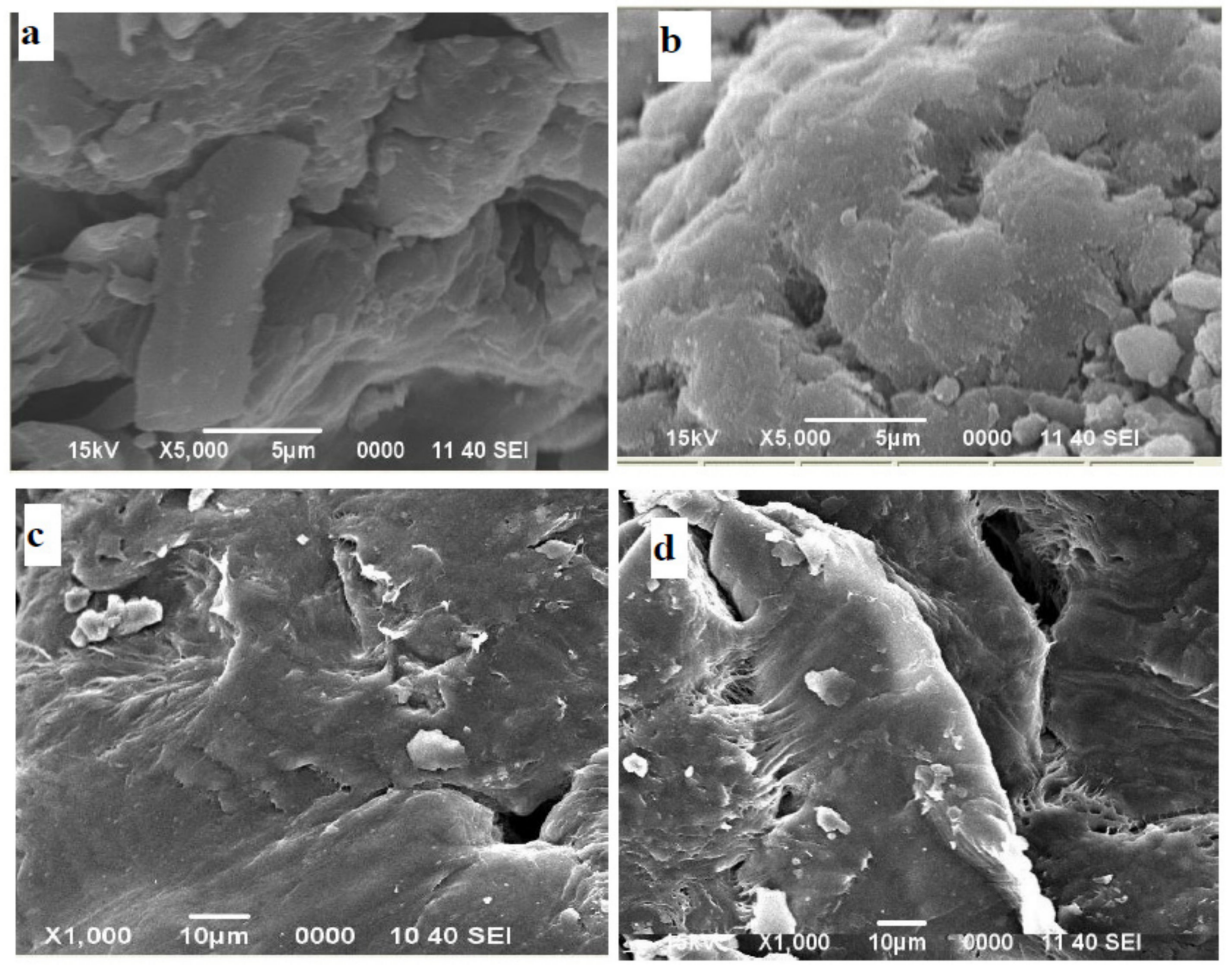
Images of (**a**) PANI (**b**) PEO (**c**) PANI-PEO-KNO_3_and (**d**) PANI-PEO-NaNO_3_.

Figure 3 shows the scanning electron micrographs of PANI, PEO, and its composites. The SEM micrograph of pure PANI (Figure 3a) shows rough and high agglomerated pellet like polycrystalline morphology [21]. The irregular surface morphology of PANI is due to the rapid growth of aniline monomers before the symmetric organization and asymmetric association of phenazine nucleates. The morphology can also be affected by greater number of entangled molecules during aggregation [22]. 

Figure 3b corresponds to micrograph of PEO which shows rough morphology of PEO as reported in literature [23]. The SEM images of PANI-PEO-KNO_3_ and PANI-PEO-NaNO_3_ composites are shown in Figure 3c,d, respectively. These images show the association of the cations of sodium and potassium to a small extent due to their dissimilar nature. However, the incorporation of potassium and sodium salts converts the composites into a gel like material. This makes the composite materials more flexible and stiff [24]. The pores and cracks in figure c and d may be due to the heat treatment during drying of the samples. However, such a porous surface morphology is best suited for adsorption phenomena.

### 3.4. XRD Analysis

**Figure 4:**
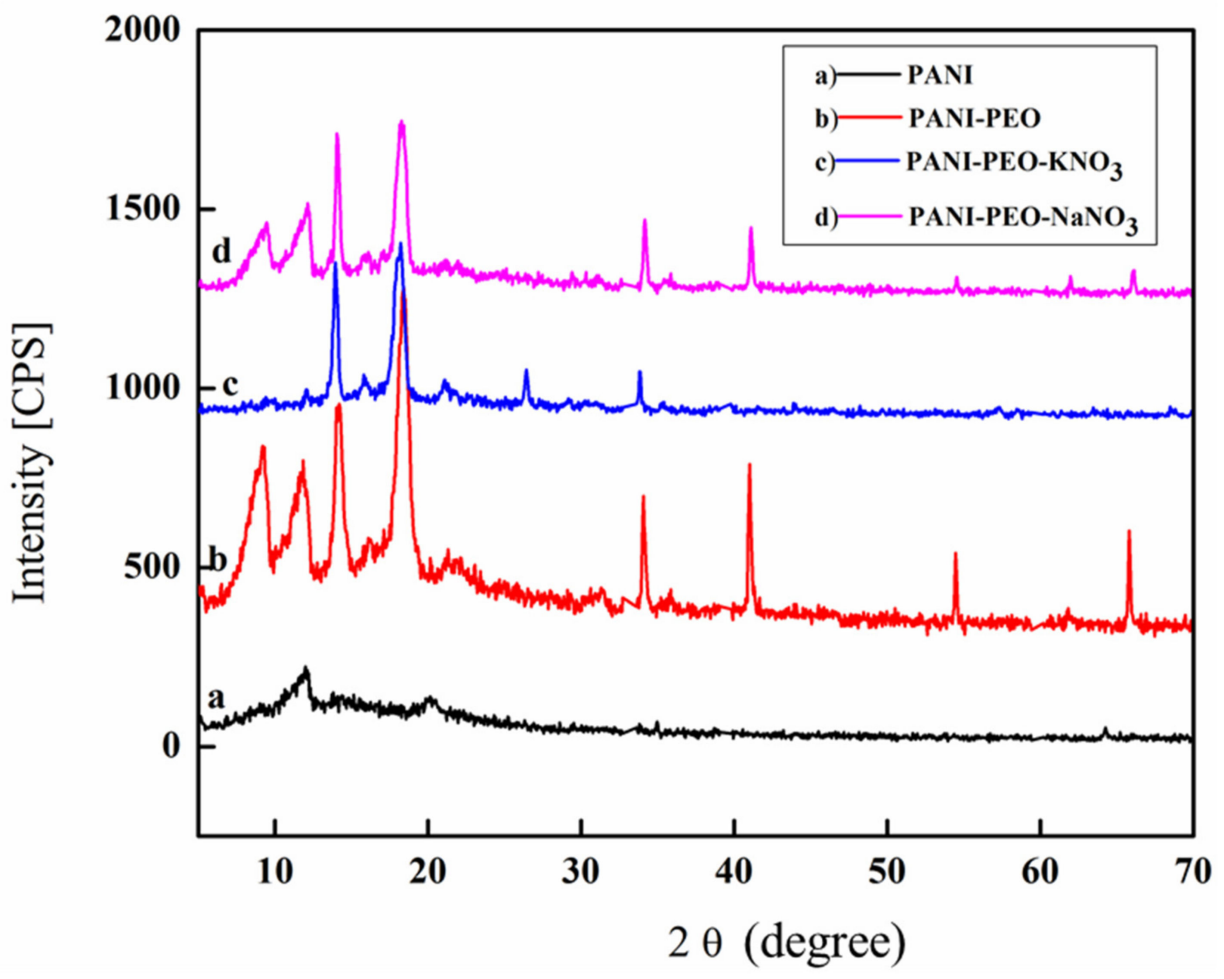
XRD patterns of (**a**) PANI (**b**) PANI-PEO (**c**) PANI-PEO-KNO_3_ (**d**) PANI-PEO-NaNO_3_.

Figure 4a shows the XRD pattern of PANI which represents extensive elevated angle asymmetric peak scattering at 2θ stuck between 12–20°. The respective climax cover one broad peak located around 19.28° equivalents to d-spacing of 3.5256 Å, identifying a small level of crystallinity [25]. XRD patterns of PANI-PEO blend and its composites with KNO_3_ and NaNO_3_ salts are shown in Figure 4b–d, respectively. The two distinctive peaks of polymeric blend are found between 2θ 16.1° and 20.3° which are the characteristic peaks of PEO and PANI. The decrease broadness in intensity of the corresponding peaks is observed in case of PANI-PEO blend [26]. This might be due to the disruption of crystalline structure of PEO by PANI. PANI-PEO-KNO_3_ composite shows further decrease in crystallinity which might be due to greater ionic size of potassium (K^+1^) causing more dispersion of PANI-PEO chain in the composite. On the other hand Na^+^ ionic size are much smaller relative to K^+^, which does not affect the interaction between PANI and PEO chains like potassium. This fact can further be demonstrated by the increased crystallinity of PANI-PEO and PANI-PEO-NaNO_3_ [27].

### 3.5. Rheological Study

Rheological properties are very important as they give information about good mixing and miscibility of polymeric materials and their components. The rheological properties of synthesized, PANI, PANI-PEO blend and PANI-PEO-KNO_3_ and PANI-PEO-NaNO_3_ composites were studied in order to understand their flow behavior.

Maximum care was undertaken in selection of solvents and other parameters for fabrication of composites and to perform the rheological measurements. It is worth noting that pyridine and chloroform are used for a lot of reactions. Chloroform clearly has a tendency of higher evaporation rate at lower boiling point. However, the nitrogen lone pair in pyridine is very exposed and easily can make a hydrogen bond complex with chloroform and other haloforms which decrease the rate of evaporation extensively. 

**Figure 5:**
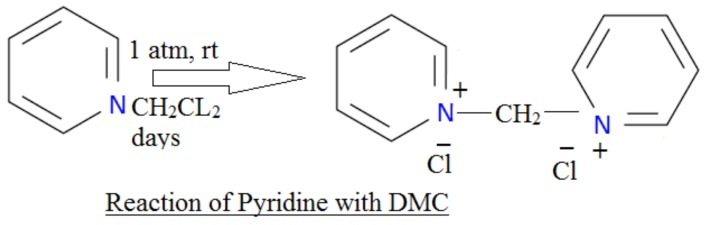
Representation of reaction of pyridine with DMC.

The mechanistic approach of pyridine with dichloromethane, DMC, proposed by Alexander et al. as shown below in Figure 5 [27].

**Figure 6:**
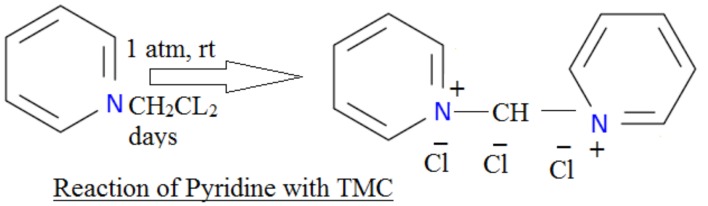
Representation of reaction of pyridine with TMC (choloroform).

Same is the case with Trichloromethane, TMC, (chloroform) as shown below in Figure 6.

We had observed that the sample retained its jelly like texture and did not become solid film at room temperatures that shows resistance of solvents to further vaporization from the samples at room temperature. Katugampola and Winstead declared that pyridine is the best solvent for rheological measurements [28].

The boiling points of pyridine and chloroform are 115.3 °C and 61.2 °C respectively, which are much higher as compared to the temperature (20 °C) of the composites during rheological measurements. Additionally, “Anton Paar Rheometer Physica MCR 301” used in this study have paltier controlled temperature system which do not vaporization of the solvent contents while performing rheological analysis.

#### 3.5.1. Flow Curves

**Figure 7:**
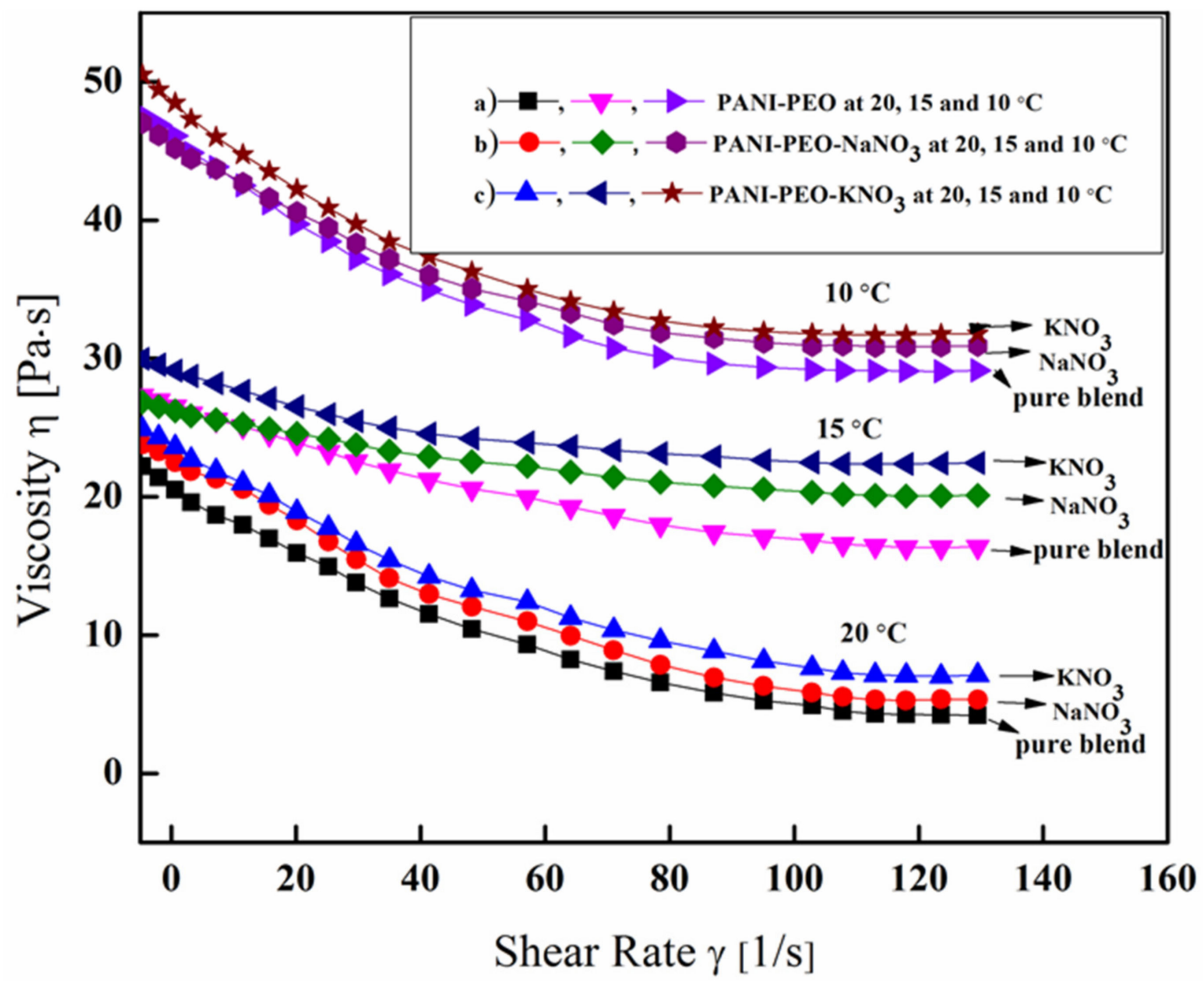
Dependence of viscosity of (**a**) PANI-PEO pure blend (**b**) PANI-PEO-NaNO_3_ (**c**) PANI-PEO-KNO_3_ at 20 °C, 15 °C and 10 °C.

The flow curves of log viscosity versus log shear rate of PANI-PEO blend, PANI-PEO-KNO_3_ and PANI-PEO-NaNO_3_ composites were recorded at 10 °C, 15 °C and 20 °C as shown in Figure 7. The shear rate and temperature applied for rheological measurement shown in Figure 7 has inverse relation with viscosity of the polymer composites. 

It is evident that viscosity of PANI-PEO blend is continuously decreasing with increasing shear rate at all temperatures. The non-linear relation of viscosity with shear rate was observed which clarify shear thinning and non-Newtonian behavior. Generally, polymers having sufficiently high molecular weights are always entangled (like spaghetti) and randomly oriented when at rest. However, when sheared, they begin to disentangle and to align, resulting in the drop of viscosity. The degree of disentanglement will depend on the shear rate. At sufficiently high shear rates, the polymers will be completely disentangled and fully aligned. The observed decrease in viscosity and shear thinning effect might also be due to the weakening of inter and intra chain interactions which result in sample deformation. Apart from this phenomenon of disentanglement, several factors such as threshold percolation shear rate, shear stress, temperature, concentrations, particle size, particle shape, and applied external load effecting the orientation and surface deformation of polymeric composites can be responsible for shear thinning and non-Newtonian fluid behavior. 

The FTIR, XRD studies as discussed above reveal the complete miscibility of salts into PANI-PEO matrices. However, as both salts have different ionic sizes, so the behavior towards shear rate is also somewhat different. Initially, at lower shear rates, the rate of decrease in viscosity is less while at higher shear rates, the viscosity decreases much faster.

In Figure 7b,c, constant viscosity regime is observed at higher shear rate. It is assumed that at low shear rates, the interactions among inorganic salts ions cannot be disintegrated and thus it is hard to flow. However, with increasing shear rate (γ) the inter atomic interaction becomes weak and as a result structures break down and deformation arises that ultimately results in the decrease in viscosity. It should be noted in Figure 7 that rheological measurements become difficult for (γ) above 130 s^−1^ due to the formation of macroscopic aggregates and instabilities in the composites. The effect of inorganic salts on decline viscosity of PEO based composites are reported in literature [29]. 

The fillers and inorganic salts (e.g., KNO_3_ and NaNO_3_) added to PEO based composite tend to reduce the hydrodynamic volume of the polymer molecule, which results in the decline of intrinsic viscosity of the composite [30]. Similarly, the particle size, shape and particle distribution have greater effect on the viscosity of the polymeric composite. As the cationic and ionic size of KNO_3_ and NaNO_3_ are small, so these ions move freely without facing any hindrance [31]. It can be seen from our results that pure blend PANI-PEO has lower viscosity at all temperatures. But when inorganic salts are added, the composites show somewhat higher values of viscosity. The resistance to flow has somewhat increased with addition of salts in composites in comparison to pure PANI-PEO blend. As a whole, shear thinning behavior is observed by all these. The temperature change has profound effect on shear viscosity. It can be seen that at low temperature i.e., 10 °C pure PANI-PEO has viscosity ≈ 46 Pa·s while PANI-PEO-KNO_3_ composite has ≈ 51 Pa·s which at 20 °C drops to 22 and 25 Pa·s, respectively. This effect shows that temperature must be considered seriously before making the use of these materials, although these may be feasible materials for screen printing.

#### 3.5.2. Frequency Sweep Tests

Frequency sweep tests are carried out to analyze the storage modulus (G′) and loss modulus (G″) as a function of angular frequency (ω) for PANI blend and its composites at different temperatures. The storage modulus concerned with the measurement of stored energy suggests elastic behavior, while the loss modulus is to that of energy dissipation per capita of sinusoidal deformation corresponding to the viscous behaviors of the material.

**Figure 8:**
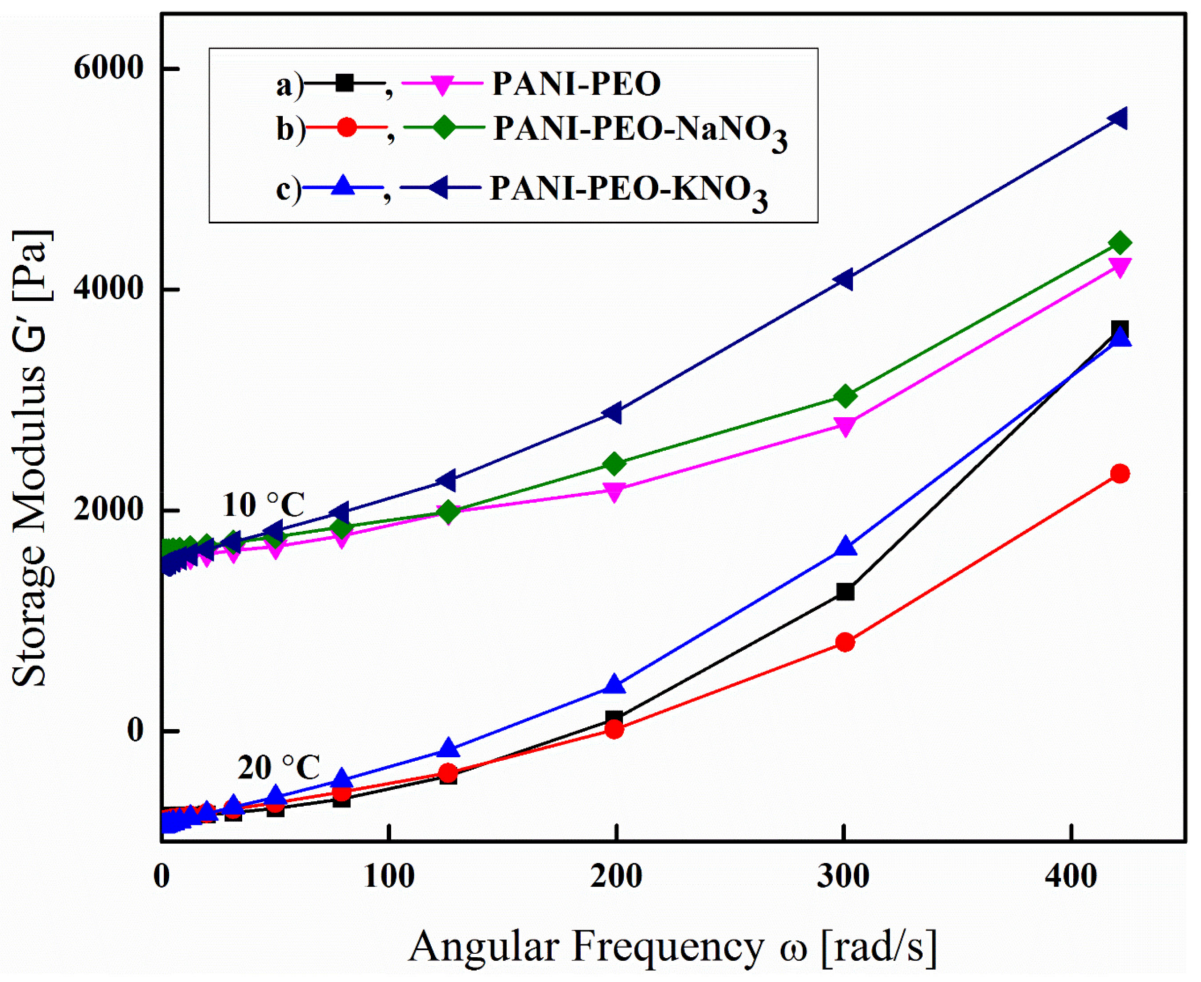
Rheogram of (**a**) PANI-PEO (**b**) PANI-PEO-NaNO_3_ and (**c**) PANI-PEO-KNO_3_ indicating dependence of storage modulus on angular frequency at 20 °C, and 10 °C.

**Figure 9:**
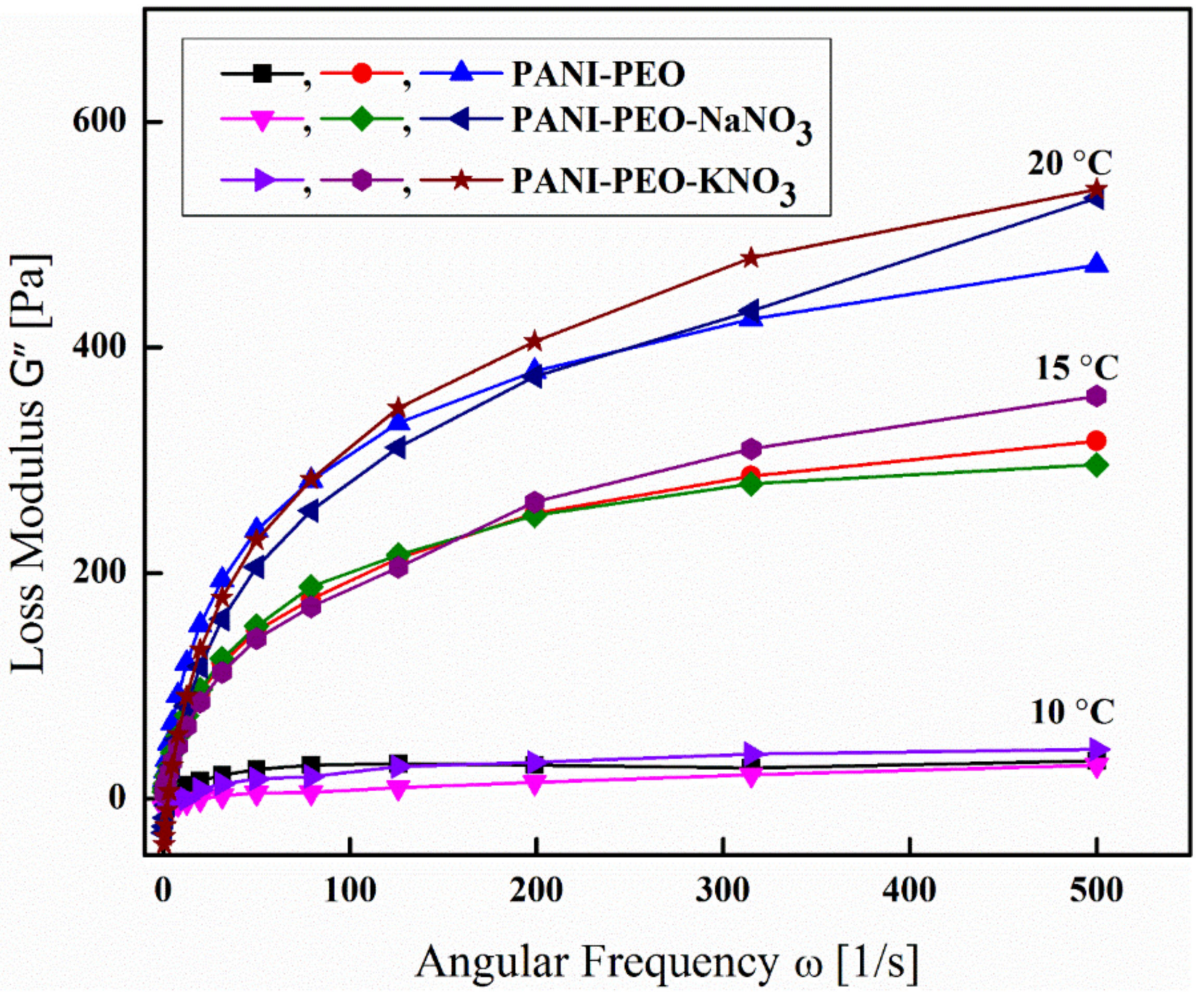
Rheogram of (**a**) PANI-PEO (**b**) PANI-PEO-NaNO_3_ and (**c**) PANI-PEO-KNO_3_ showing dependence of loss modulus on angular frequency at 20 °C, 15 °C and 10 °C.

Figure 8 and Figure 9 demonstrates the relationship of storage modulus (G′) and loss modulus (G″) versus angular frequency at different temperatures, respectively. It is clear from the Figure 8 and Figure 9 that the storage modulus and loss modulus values are higher at an elevated frequency range for all testing samples. However, storage modulus has inverse while loss modulus has direct relation with temperature. Furthermore PANI-PEO-KNO_3_ has greater G′ and G″ values than PANI-PEO blend and PANI-PEO-NaNO_3_ composite. These results suggested a strong interaction among polymers and salt particles, which specify excellent elasticity. 

The improvement in elasticity is because of strong cross-linking of the phenyl ring of PANI in PEO backbone. This illustration reveals better stiffness which confirms the stability of the blend and composites. From Figure 8 it is seen that the storage modulus (G′) increases with increase in angular frequency (ω) and reaches up to about 6000 Pa. Materials with such a high storage modulus (G′) values represent their capacity to store the applied stress (energy) and hence such materials could be best suited for application in mechanically tough membranes.

In the similar way, comparison of G′ and G″ values shows that G′ values are much higher and loss factor tan σ (tan σ = G″/G′) values are less than 1. This also confirms the elastic behavior of the system. 

**Figure 10:**
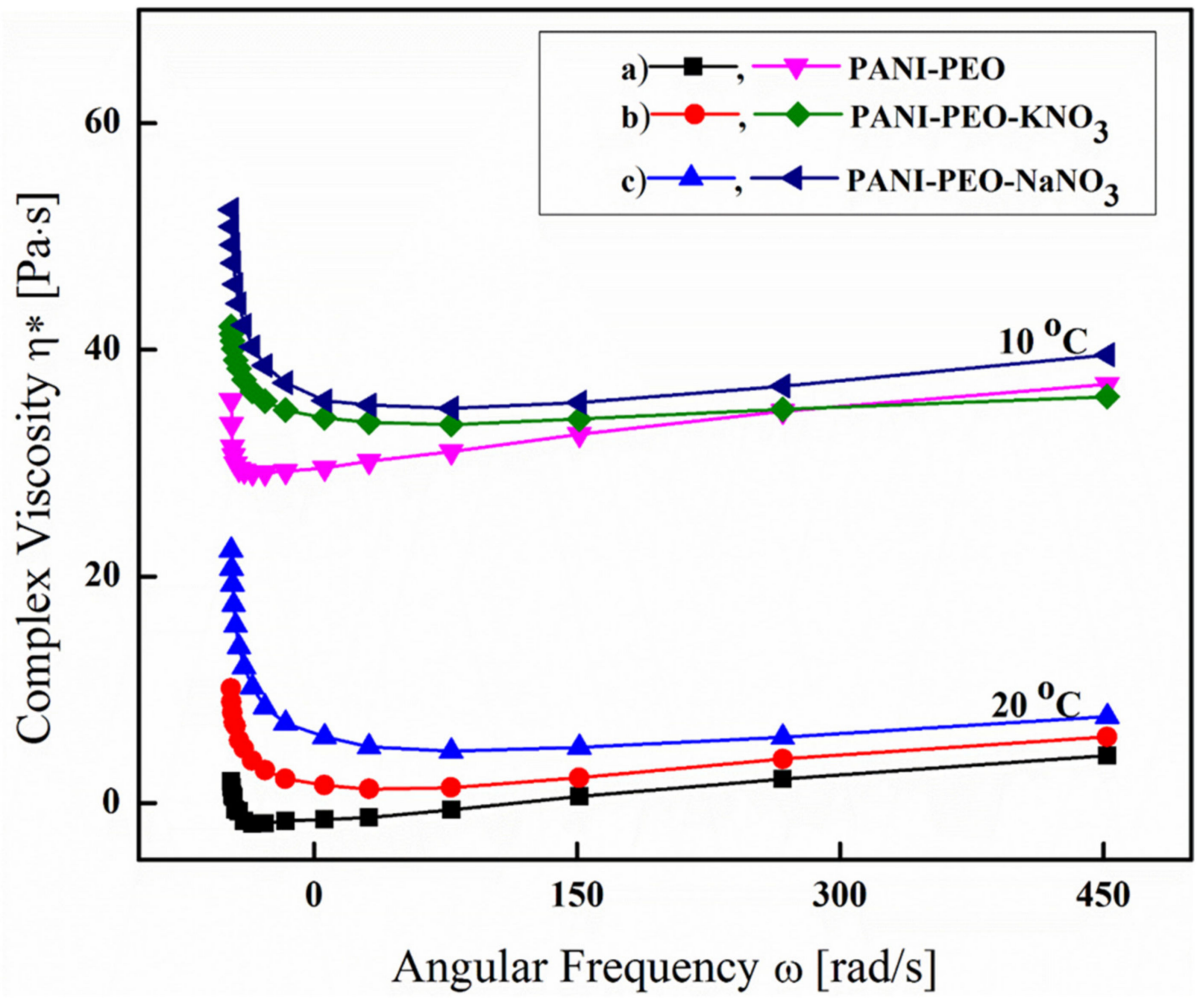
Rheogram of (**a**) PANI-PEO (**b**) PANI-PEO-NaNO_3_ and (**c**) PANI-PEO-KNO_3_ depicting dependence of complex viscosity on angular frequency at 10 °C and 20 °C.

The flow and viscoplastic behavior of the PANI-PEO composites are demonstrated from the rheogram of angular frequency vs complex viscosity shown in Figure 10. The yield stress shown in frequency sweep tests is less than a like true yield. This may be because of reducing hydrodynamic volume of the polymer molecules and entanglement of the system. The material appears to have a yield stress but depicted at much lower shear rates. However, such systems could possibly be used in different electronic and energy storage devices because of its high storage capacity.

In Figure 10, a steady drop off in complex viscosity (η*) with respect to angular frequency (ω) is observed in all testing samples, which imply a shear thinning behavior. However, it is interesting that when angular frequency increases above 30 rad/s the complex viscosity converge towards the same line where no further decrease occurs in η*. The drop off in complex viscosity with frequency sweep is because of the arrangement of nitrate ions and aniline monomers in PEO matrix. The nitrate ions of sodium and potassium have great impact on the viscosity and tend to minimize this effect and finally become aligned which no further change in viscosity occurs. The same trend was reported by Volk and his co-workers [31].

#### 3.5.3. Rheological Testing Models

**Figure 11:**
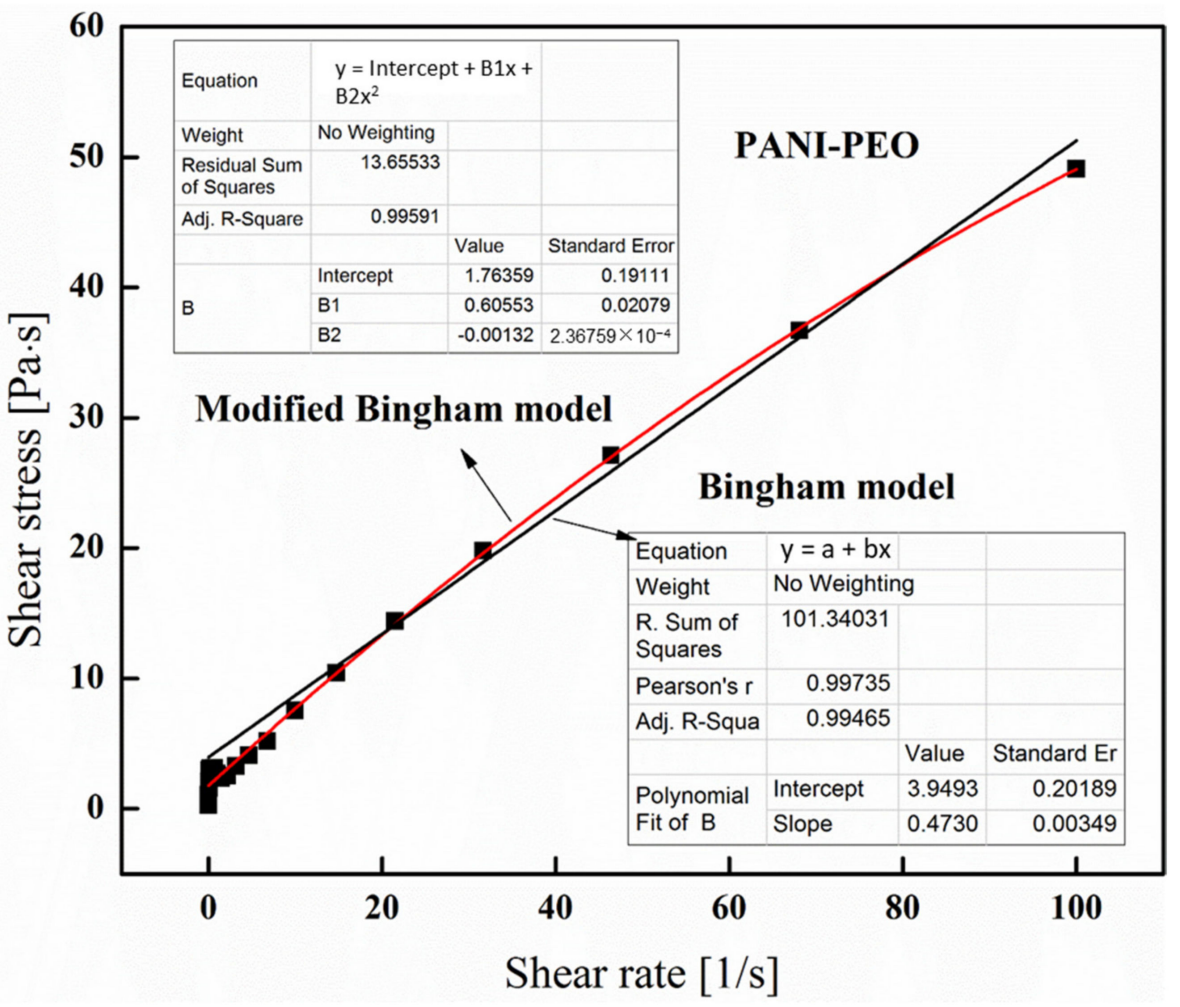
Bingham and modified Bingham models for PANI-PEO blend at room temperature.

**Figure 12:**
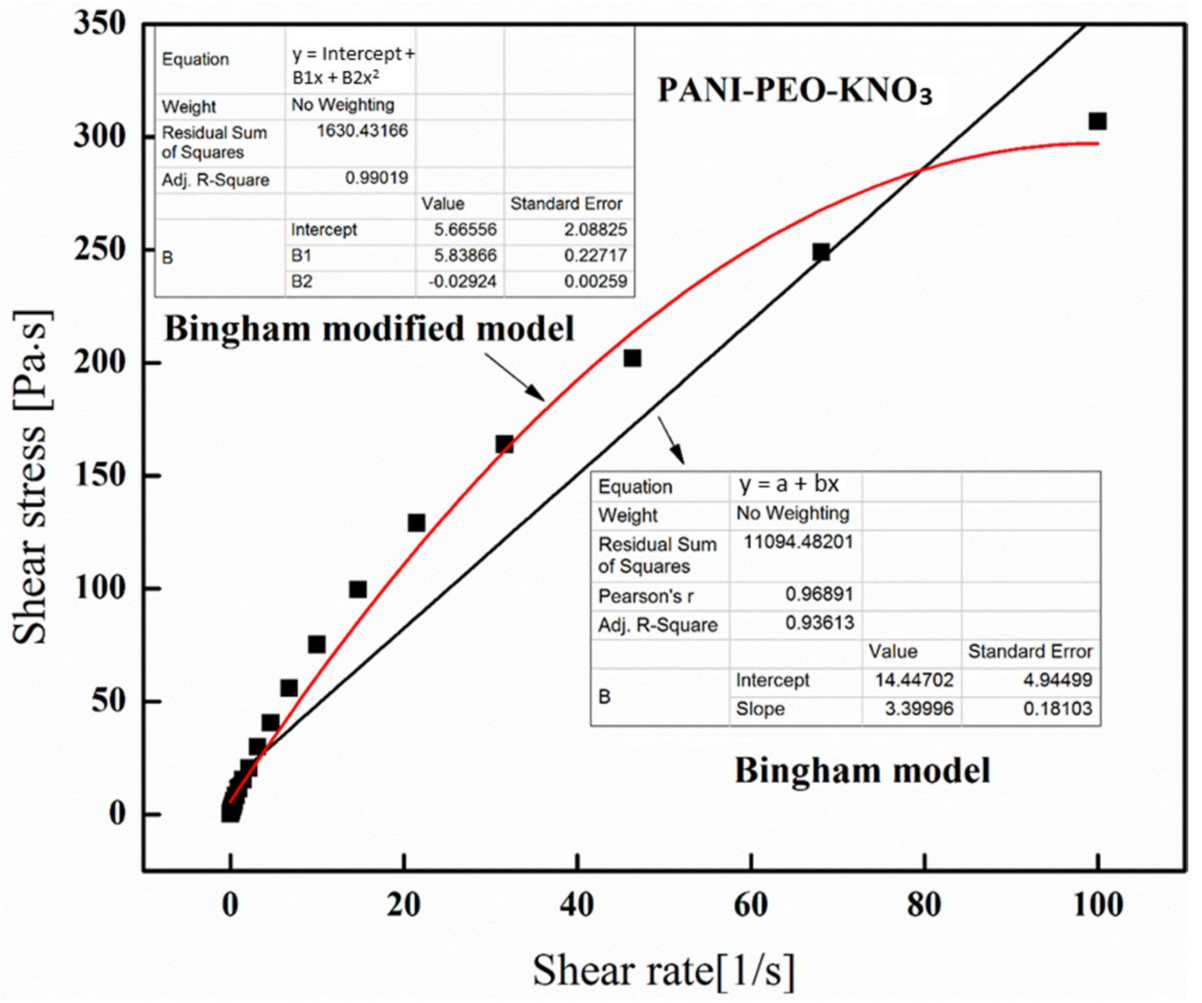
Bingham and modified Bingham models for PANI-PEO-KNO_3_ composite at room temperature.

The fluid behavior of the synthesized samples were further confirmed by Bingham (τ = τ_y_ + m_B_γ) and modified Bingham (τ = τ_y_ + _B_γ + Cγ^2^) as the first approximation model and are presented in Figure 11 and Figure 12. Where τ is the shear stress, τ_y_ is yield stress, m_B_ Bingham plastic viscosity and γ is the shear rate.

Both Bingham models verified the non-Newtonian and shear thinning behaviors of the samples by mean of its regression coefficient (R^2^) values for PANI-PEO blend and composites as shown in Figure 11 and Figure 12, respectively. The R^2^ value further verified that Bingham modified model is best fitted for both blend and composite. PANI-PEO has a stress yield of 3.9493 Pa·s while in case of PANI-PEO-KNO_3_ the stress yield is 14.44702 Pa·s, affirming that the stress yield of the composite is much higher than pure blend.

This suggests that inorganic salt particles have some kind of interaction with large polymer molecules creating weak solid structure. So, more stress is required to brook these structures. Once these structures are broken, then the system particles move within the liquid under viscous force. This is reversible i.e. if stress is removed, then the structure may form again.

## 4. Conclusions

The PANI-PEO and their composites with nitrate salts of Na and K can be synthesized via solution casting techniques without using any additives, fibers or plasticizers, once a PANI soluble in chloroform or other suitable solvent is obtained. The complexation of PANI with PEO, NaNO_3_, and KNO_3_ can lead to better mechanical properties in comparison with PANI. The synthesized composite materials showed good thermal stability, well elasticity, and high capability of storage. The presented procedure for the formation of composites is simple and facile and rheological studies suggest that it can be used as an effective way to improve the some of the desired properties of PANI based materials.

## Figures and Tables

**Table 1:** Stability of PANI, PEO and its composites.

Material	Temperature 1 (°C)	Temperature 2 (°C)	Carbonized Mass (%)
PANI	195	320	20
PEO	192	360	08
PANI-PEO-NaNO_3_	250	350	06
PANI-PEO-KNO_3_	240	380	08
